# Supplementary material for: CELLama: Foundation Model for Single Cell and Spatial Transcriptomics by Cell Embedding Leveraging Language Model Abilities
Source: Adv Sci (Weinh). 2025 Nov 17;13(7):e13210. doi: 10.1002/advs.202513210 (PMC12866849; doi:10.1002/advs.202513210)
Supplement: Supplementary file 1 — Supporting Information [file ADVS-13-e13210-s002.pdf]

Supplementary Figures

CELLama: Foundation Model for Single Cell and Spatial Transcriptomics by Cell Embedding Leveraging Language Model Abilities

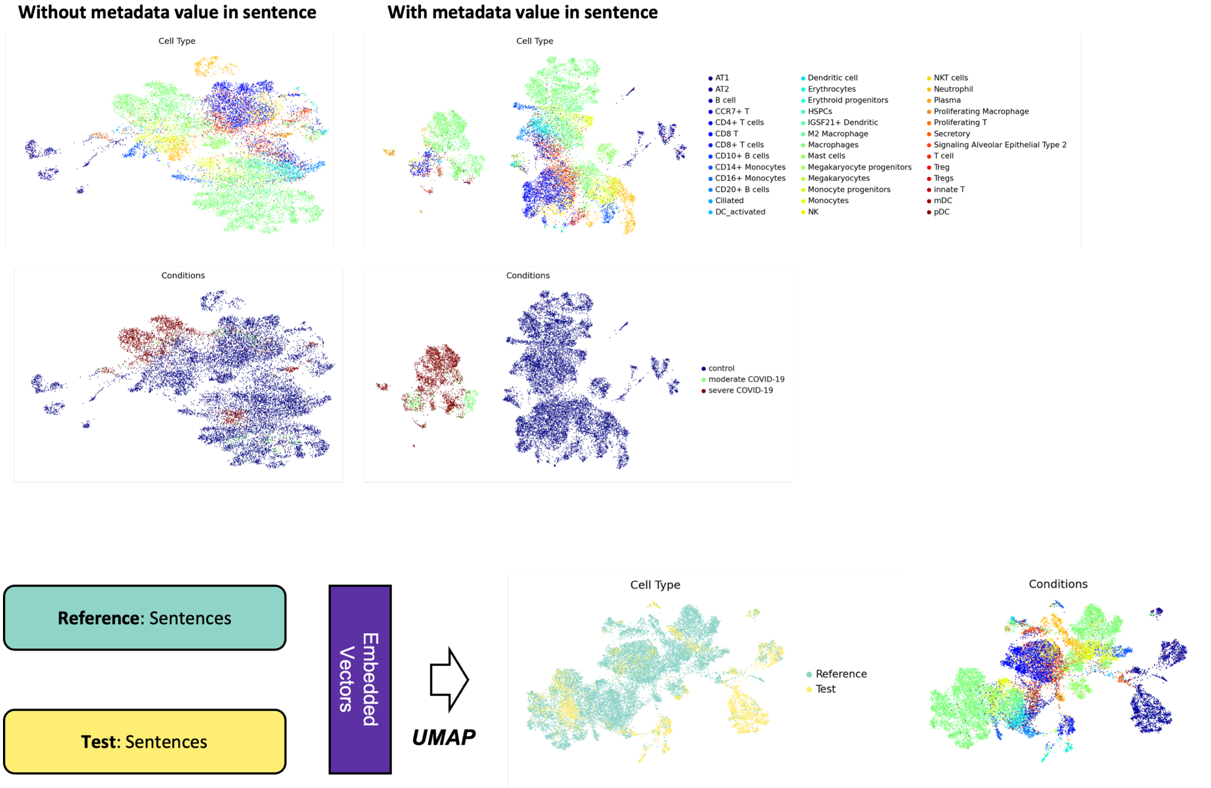

Supplementary Figure 1. CELLama embedding for the COVID-19 scRNA-seq dataset. Visualization of the embedding variability in the COVID-19 dataset based on the inclusion of metadata. The dataset consisted of 18 distinct batches from lung tissues, highlighting the adaptability of embedding under different metadata conditions. The reference and test sets were segmented and subsequently embedded into a unified data space, which was visualized using UMAP.

a

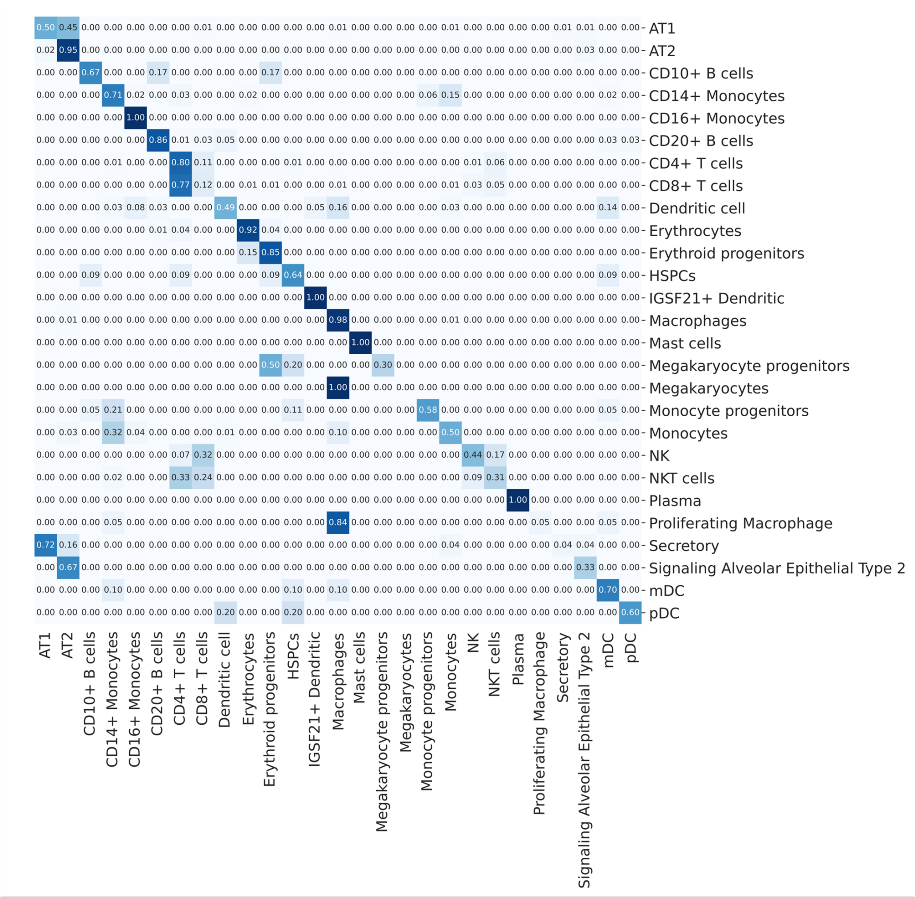

b

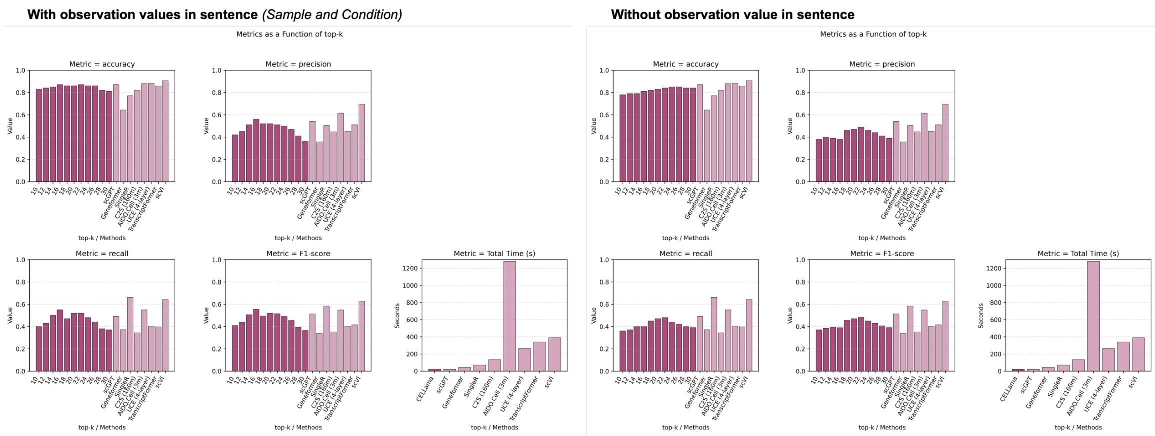

Supplementary Figure 2. Comparison of cell type mapping using CELLama and ground truth labels of COVID-19 dataset.

(a) The confusion matrix illustrates the performance of CELLama in classifying cell types against verified labels, providing a direct comparison to evaluate the efficacy of zero-shot learning scenarios.

(b) Performance metrics of CELLama in cell typing using a general-purpose sentence transformer and other methods: scGPT, Geneformer, SingleR, C2S (160m), AIDO-Cell (3m), UCE (4-layer), TranscriptFormer (TF-Sapiens), and scVI. The results for C2S and TranscriptFormer are consistently shown with metadata involved in embedding. CELLama with a general-purpose sentence transformer model, *all-MiniLM-L12-v2*, achieved slightly better precision and recall than scGPT and Geneformer. The accuracy of CELLama was lower than scGPT when metadata was not included, underscoring the influence of metadata on CELLama classification ability. Also, CELLama operates on a time scale comparable to scGPT and Geneformer, whereas the other methods

exhibit significantly higher time complexity, limiting their scalable analysis. The raw benchmarking data can be found in **Supplementary Table S3**.

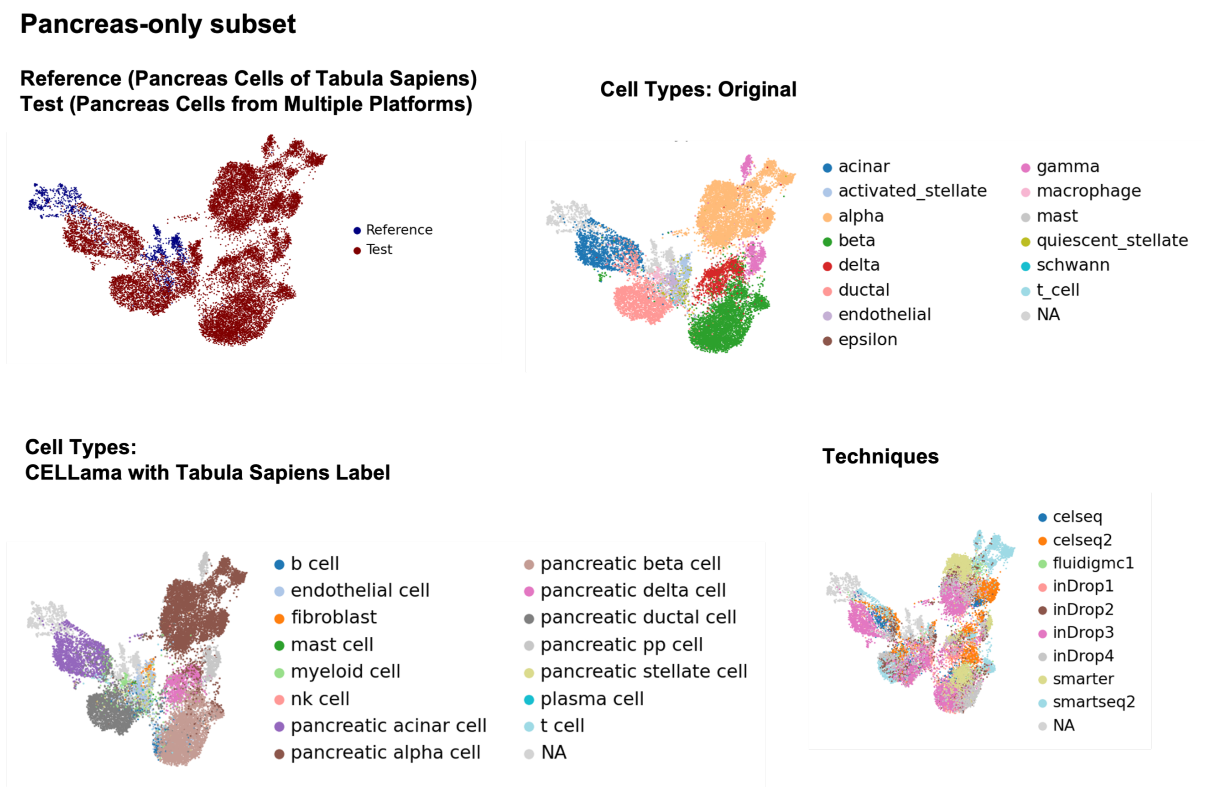

Supplementary Figure 3. Co-visualization of human pancreas scRNA-seq data and subset of pancreas cell data of Tabula Sapiens. This figure complements the primary analysis shown in **Figure 3c** by detailing how CELLama integration strategy enables alignment of the query dataset with the pancreas tissue cell data from the Tabula Sapiens reference, highlighting the robustness of CELLama in managing diverse data sources and multiple tissue-based atlas.

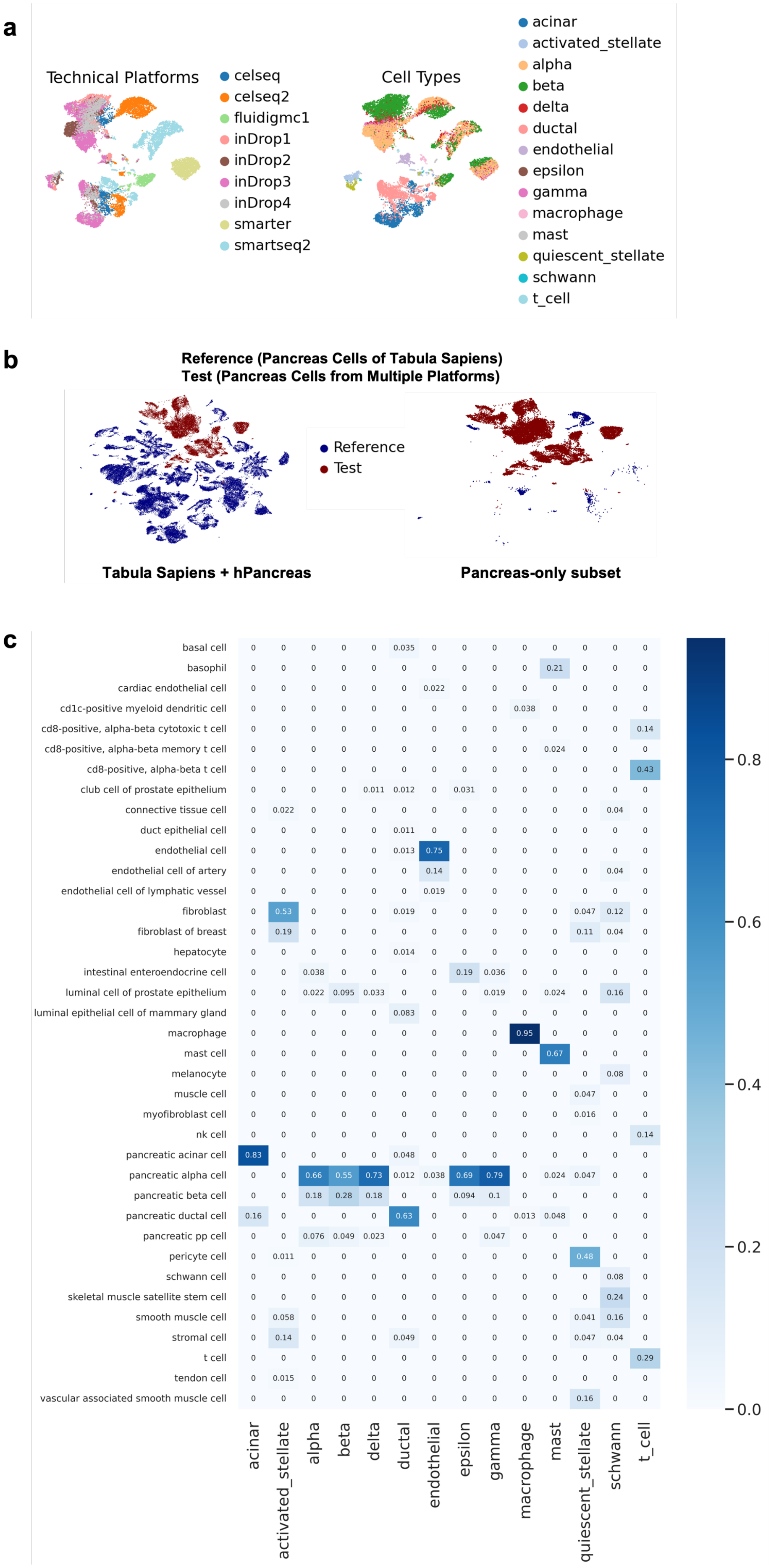
a
Technical Platforms
celseq
celseq2
fluidigmc1
inDrop1
inDrop2
inDrop3
inDrop4
smarter
smartseq2
Cell Types
acinar
activated_stellate
alpha
beta
delta
ductal
endothelial
epsilon
gamma
macrophage
mast
quiescent_stellate
schwann
t_cell
b
Reference (Pancreas Cells of Tabula Sapiens)
Test (Pancreas Cells from Multiple Platforms)
Reference
Test
Tabula Sapiens + hPancreas
Pancreas-only subset
c
basal cell 0 0 0 0 0 0.035 0 0 0 0 0 0 0 0
basophil 0 0 0 0 0 0 0 0 0 0 0.21 0 0 0
cardiac endothelial cell 0 0 0 0 0 0 0.022 0 0 0 0 0 0 0
cd1c-positive myeloid dendritic cell 0 0 0 0 0 0 0 0 0 0.038 0 0 0 0
cd8-positive, alpha-beta cytotoxic t cell 0 0 0 0 0 0 0 0 0 0 0 0 0 0.14
cd8-positive, alpha-beta memory t cell 0 0 0 0 0 0 0 0 0 0 0.024 0 0 0
cd8-positive, alpha-beta t cell 0 0 0 0 0 0 0 0 0 0 0 0 0 0.43
club cell of prostate epithelium 0 0 0 0 0.011 0.012 0 0.031 0 0 0 0 0 0
connective tissue cell 0 0.022 0 0 0 0 0 0 0 0 0 0 0.04 0
duct epithelial cell 0 0 0 0 0 0.011 0 0 0 0 0 0 0 0
endothelial cell 0 0 0 0 0 0.013 0.75 0 0 0 0 0 0 0
endothelial cell of artery 0 0 0 0 0 0 0.14 0 0 0 0 0 0.04 0
endothelial cell of lymphatic vessel 0 0 0 0 0 0 0.019 0 0 0 0 0 0 0
fibroblast 0 0.53 0 0 0 0.019 0 0 0 0 0 0.047 0.12 0
fibroblast of breast 0 0.19 0 0 0 0 0 0 0 0 0 0.11 0.04 0
hepatocyte 0 0 0 0 0 0.014 0 0 0 0 0 0 0 0
intestinal enteroendocrine cell 0 0 0.038 0 0 0 0 0.19 0.036 0 0 0 0 0
luminal cell of prostate epithelium 0 0 0.022 0.095 0.033 0 0 0 0.019 0 0.024 0 0.16 0
luminal epithelial cell of mammary gland 0 0 0 0 0 0.083 0 0 0 0 0 0 0 0
macrophage 0 0 0 0 0 0 0 0 0 0.95 0 0 0 0
mast cell 0 0 0 0 0 0 0 0 0 0 0.67 0 0 0
melanocyte 0 0 0 0 0 0 0 0 0 0 0 0 0.08 0
muscle cell 0 0 0 0 0 0 0 0 0 0 0 0.047 0 0
myofibroblast cell 0 0 0 0 0 0 0 0 0 0 0 0.016 0 0
nk cell 0 0 0 0 0 0 0 0 0 0 0 0 0 0.14
pancreatic acinar cell 0.83 0 0 0 0 0.048 0 0 0 0 0 0 0 0
pancreatic alpha cell 0 0 0.66 0.55 0.73 0.012 0.038 0.69 0.79 0 0.024 0.047 0 0
pancreatic beta cell 0 0 0.18 0.28 0.18 0 0 0.094 0.1 0 0 0 0 0
pancreatic ductal cell 0.16 0 0 0 0 0.63 0 0 0 0.013 0.048 0 0 0
pancreatic pp cell 0 0 0.076 0.049 0.023 0 0 0 0.047 0 0 0 0 0
pericyte cell 0 0.011 0 0 0 0 0 0 0 0 0 0.48 0 0
schwann cell 0 0 0 0 0 0 0 0 0 0 0 0 0.08 0
skeletal muscle satellite stem cell 0 0 0 0 0 0 0 0 0 0 0 0 0.24 0
smooth muscle cell 0 0.058 0 0 0 0 0 0 0 0 0 0.041 0.16 0
stromal cell 0 0.14 0 0 0 0.049 0 0 0 0 0 0.047 0.04 0
t cell 0 0 0 0 0 0 0 0 0 0 0 0 0 0.29
tendon cell 0 0.015 0 0 0 0 0 0 0 0 0 0 0 0
vascular associated smooth muscle cell 0 0 0 0 0 0 0 0 0 0 0 0.16 0 0
acinar
activated_stellate
alpha
beta
delta
ductal
endothelial
epsilon
gamma
macrophage
mast
quiescent_stellate
schwann
t_cell
0.8
0.6
0.4
0.2
0.0

Supplementary Figure 4. scGPT embeddings for human pancreas data across multiple platforms and their mapping to a general reference atlas, Tabula Sapiens.

(a) scGPT-embedded representation of human pancreas single-cell data from multiple platforms, showing clustering primarily by cell type. However, one batch, smarter, formed a distinct cluster, suggesting batch effects in the embedding.

(b) scGPT-embedded pancreas data mapped onto a general-purpose reference atlas (Tabula Sapiens). Human pancreas cells from Tabula Sapiens data were distinctly separated from pancreas cells obtained from other platforms, indicating challenges in cross-dataset alignment when using a general multi-tissue reference.

(c) Zero-shot cell typing performance using the general reference dataset of scGPT. It demonstrates difficulties in identifying rare cell types, for instance, beta cell annotation achieved 28% accuracy, while delta cells were identified at less than 1% accuracy.

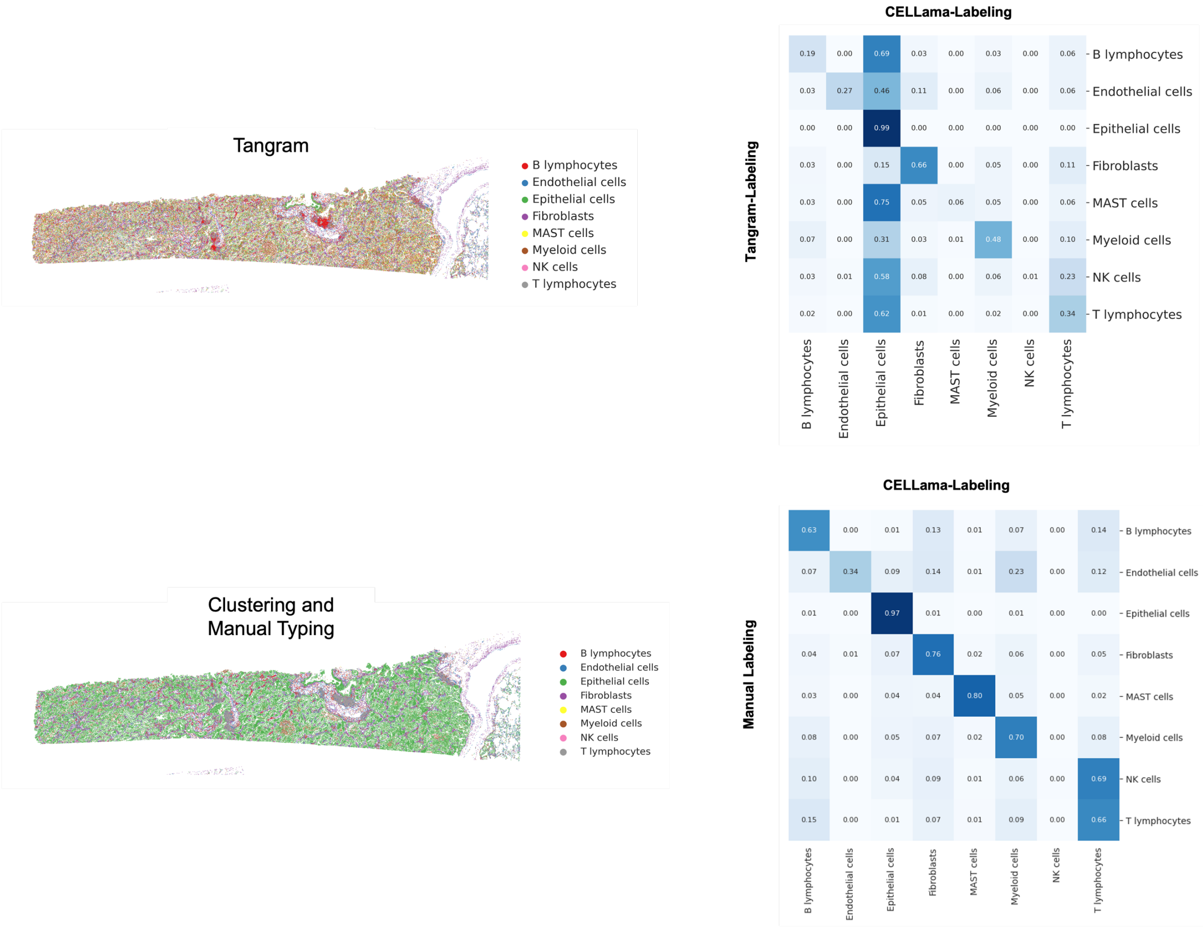

Supplementary Figure 5. Cell typing comparison using CELLama and other methods

We compared cell typing results obtained from CELLama, Tangram, and manual labeling after clustering. Canonical marker gene expression levels were assessed, and clusters were manually annotated based on these markers. The comparison revealed that CELLama-based labeling closely matched manual annotations, while Tangram identified relatively fewer cancer epithelial cells.

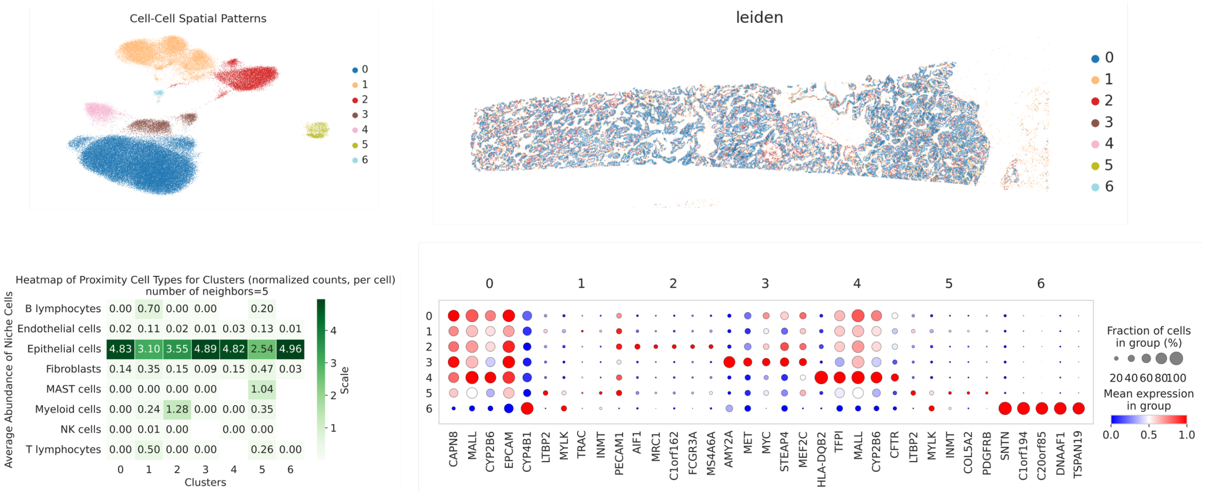

Supplementary Figure 6. Niche-based subtyping for epithelial cells using CELLama

We extended the spatial mapping analysis of CELLama to epithelial cells within the same ST data of **Figure 6**. Following the methodological approach outlined for fibroblasts, epithelial cells were also analyzed for niche-specific patterns and clustering based on proximity to other cell types. CELLama was employed to generate embeddings that incorporate niche cell type information, enabling the visualization of subclusters within the epithelial population (*upper left*). This subclusters of epithelial cells were visualized on the spatial map (*upper right*). Each subcluster showed different patterns of niche cell types (*lower left*) and markers (*lower right*) could be obtained by differentially expressed genes of each subcluster.

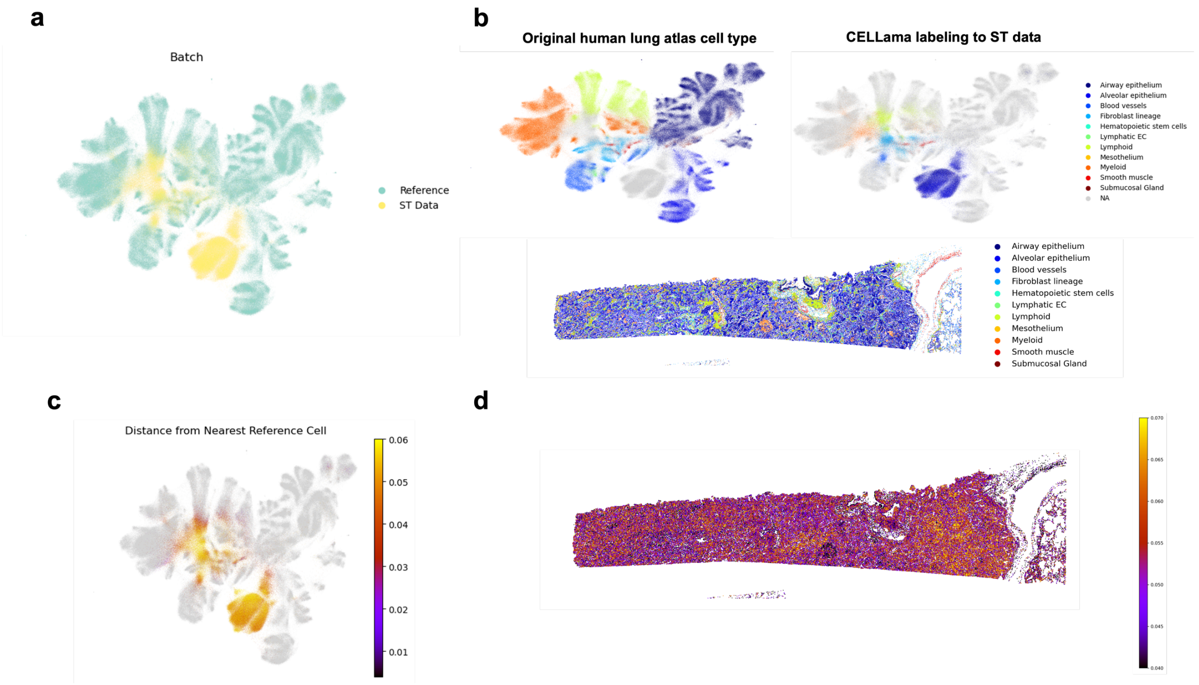

Supplementary Figure 7. CELLama embedding using normal lung cell atlas to ST data of lung cancer.

(a) Even though ST data of lung cancer were different from normal lung cell atlas, these two datasets could be co-embedded.

(b) The embeddings facilitated the identification of the nearest cell types in the lung atlas, for instance, predominantly mapping cancerous epithelial cells to the alveolar epithelium of the normal lung.

(c) The distance maps of the embedded space generated from these CELLama provide a quantitative measure of how the query cell data (ST data of lung cancer) diverge from the normal lung cell atlas for each cell.

(d) The spatially visualized distance map of embedded space showed potential spatio-temporal trajectories and highlighting regions of pathophysiological progression. This approach underscores the potential in offering out-of-distribution measures that can inform the understanding of disease dynamics and tissue heterogeneity.

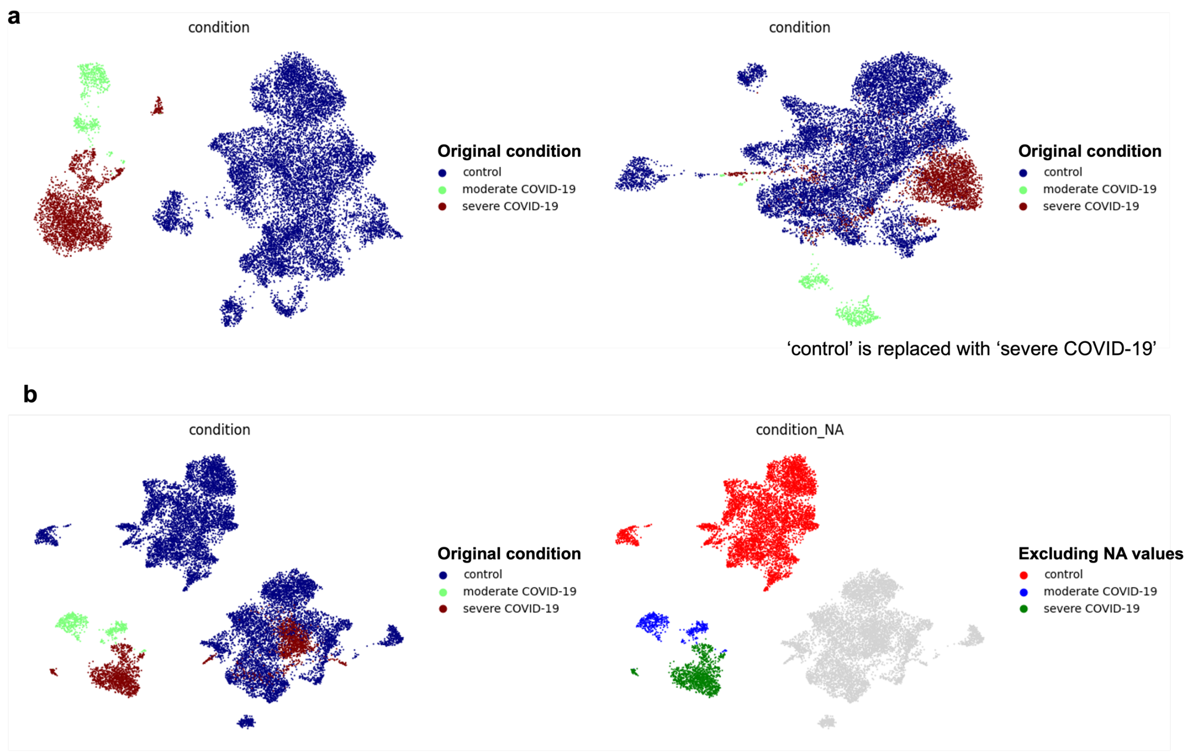

Supplementary Figure 8. Evaluation of performance degradation when the metadata is incorrect or absent.

(a) The UMAP visualization of cells colored by the original condition. When drawing the right panel image, the 'control' cells were given 'severe COVID-19' context while other cells having the corresponding condition.

(b) The UMAP visualization of cells when the metadata is partially absent. The left plot is colored according to the original condition, whereas in the right plot, half of the 'control' and 'severe COVID-19' cells were randomly reassigned to NA values.
